# Effect of the Primary Nursing Model on Self-Care Skills of Hospitalized Older Patients with Multimorbidity: A Quasi-Experimental Study

**DOI:** 10.3390/healthcare13192457

**Published:** 2025-09-27

**Authors:** Isabel Gonçalves, Sofia Almeida, Élvio Jesus, Elisabete Nunes

**Affiliations:** 1 Centre for Interdisciplinary Research in Health, Faculty of Health Sciences and Nursing, Universidade Católica Portuguesa, 1649-023 Lisbon, Portugal; ssalmeida@ucp.pt (S.A.); ejesus@ucp.pt (É.J.); 2 Hospital da Luz Lisboa, Avenida Lusíada, 100, 1500-650 Lisbon, Portugal; 3 Nursing Research, Innovation, and Development Centre of Lisbon [CIDNUR], Escola Superior de Enfermagem de Lisboa, 1600-190 Lisbon, Portugal; enunes@esel.pt

**Keywords:** multimorbidity, older patients, therapeutic self-care, primary nursing, hospital, quasi-experimental study

## Abstract

**Background/Objective**: The global increase in life expectancy has led to a higher prevalence of multimorbidity among older patients, often requiring frequent and complex healthcare. Enhancing self-care skills during hospitalization is a key priority in promoting patient autonomy and improving outcomes. The objective of the study was to analyze the effect of the primary nursing model on the therapeutic self-care of older patients with multimorbidity during hospitalization. **Methods**: It was a quasi-experimental study conducted in two comparable medical-surgical units of a private hospital in Portugal. The intervention unit adopted the primary nursing model, while the control unit maintained standard nursing care. A convenience sample of older patients with multimorbidity was recruited (n = 206; intervention group = 106, control group = 100). Therapeutic self-care was measured using the Portuguese version of the Therapeutic Self-Care Scale at admission, discharge, and follow-up. Statistical analyses included independent samples *t*-tests to assess between-group differences. **Results**: At baseline, the intervention group had significantly lower self-care scores than the control group (t_(191.045)_ = −2.24; *p* = 0.026). However, between admission and follow-up, the intervention group showed significantly greater improvements in self-care scores compared with the control group (t_(187.55)_ = 2.68; *p* = 0.008). **Conclusions**: The primary nursing care model contributed to enhanced therapeutic self-care skills in older patients with multimorbidity during and after hospitalization. Nurse managers and clinical teams can consider the primary nursing model as an effective care organization strategy to foster self-care, promote patient-centered outcomes, and improve care continuity for older patients with complex needs. **Trial Registration**: ClinicalTrials.gov Identifier: NCT06702150 (Registered 12 November 2024).

## 1. Introduction

Nurses play a pivotal role in contemporary healthcare systems, particularly amid rising demands caused by ageing populations, resource constraints, and the increasing burden of chronic and complex conditions [1]. Hospitalized patients with multimorbidity, defined as the coexistence of two or more chronic conditions [2] require comprehensive, coordinated care, making nurse-led care management models especially valuable.

Patient-centered care is central to modern nursing practice, with a strong emphasis on supporting self-care, preparing patients for discharge, and ensuring continuity of care [3]. Organizational models of nursing practice, defined as the structures and processes that guide nursing care delivery [4] can significantly influence patient outcomes, especially when designed to foster therapeutic relationships.

Historically, nursing care in hospitals has been delivered through various models, including functional nursing, team nursing, total patient care, and the -primary nurse model [5,6,7,8]. Among these, the primary nursing model stands out for promoting continuity, accountability, and patient-centered by assigning one nurse to be responsible for a patient’s care throughout their hospital stay [9]. Unlike individualized care, where different nurses assume responsibility on a shift-by-shift basis, primary nursing provides consistency and a therapeutic nurse–patient relationship [5].

Primary nursing is a patient-centered care model based on the relationship between nurse and patient [10]. The primary nurse is responsible for ensuring that decision-making is patient-centered, establishing the care plan and strategies for implementing it, and providing direct interpersonal communication with other members of the multidisciplinary team [10]. The coordination of the care plan is always of the primary nurse. They assume the patient’s care when on shift. They delegate it to other members of their team when absent.

To assess the effectiveness of such models, it is essential to identify outcomes that are sensitive to nursing interventions. Therapeutic self-care skills have been widely recognized as one such outcome, reflecting a patient’s ability to manage treatment, symptoms, and lifestyle adjustments independently [11,12].

With global life expectancy rising and the proportion of older patients increasing, hospitals are challenged by higher patient complexity, shorter lengths of stay, high readmission rates, and workforce constraints. Nurse managers must ensure safe, individualized, and outcome-focused care delivery [13,14]. In this context, the primary nursing model may serve as a viable strategy for enhancing care quality, especially for vulnerable populations such as older patients with multi-morbidity.

While previous studies have linked primary nursing to reduced adverse events, less is known about its potential to improve positive outcomes, such as enhanced self-care capabilities [15]. Implementing the primary nursing model appears to reduce infections related to venous catheters, pressure ulcers, falls, medication errors and urinary tract infections [16].

A systematic review analyzing studies that evaluated the impact of nursing care organization models in hospitals on patients’ health outcomes concluded that conclusive results could not be obtained due to the scarcity and lack of robustness of the studies [17].

Addressing this gap, the present study aimed to evaluate the effect of the primary nursing model on therapeutic self-care skills in hospitalized older patients with multimorbidity.

## 2. Materials and Methods

### 2.1. Design and Participants

This was a quasi-experimental, longitudinal study with a control group, conducted between April 2022 and August 2023 in two medical-surgical units of a private acute care hospital in Lisbon, Portugal. The hospital has 315 beds. The intervention unit received an organizational change in nursing care (implementation of the primary nursing model, see Table 1), while the control unit maintained the usual individualized care model. Both units had similar characteristics: the intervention unit had 34 beds and the control unit 35, and both admitted the same patient profiles. Each unit had a nursing team of 24 members. The study followed the Transparent Reporting of Evaluations with Nonrandomized Designs (TREND) guidelines [18]; see Table A1.

Participants were selected by convenience sampling. Inclusion criteria were as follows: (1) age ≥ 65 years (older patients); (2) diagnosis of at least two chronic conditions, one of which had to be cardiovascular disease, cancer, chronic obstructive pulmonary disease, or diabetes; (3) expected hospital stay > 48 h; and (4) cognitive ability to read, write, and understand the study information. Exclusion criteria included transfers to other units or levels of care within or outside the organization.

From 1829 eligible patients hospitalized in 2022 (980 in the intervention unit; 849 in the control), a total of 252 were recruited: 136 in the intervention group and 116 in the control group. The minimum required sample was 122 participants (61 per group), based on power analysis using G*Power 3.1, with an effect size of Cohen’s d = 0.66 [19], significance level of 0.05, and power of 0.95.

### 2.2. Intervention

The intervention was coordinated with the nurse director and head nurses. Primary nurses in the intervention unit were identified by the head nurse and invited to participate. Four training sessions on the primary nursing model were delivered by the principal investigator over four weeks (February–March 2022). Additional briefings occurred during shift handovers, supported by printed teaching materials. The model was implemented at the end of March 2022.

Patient recruitment began in both units in April 2022. Eligible patients were informed about the study and invited to participate. Those who consented signed a written informed consent form. In the intervention unit, patients were assigned to a primary nurse responsible for care throughout the hospital stay; in the control unit, care continued to be shift-based.

#### Objectives and Hypotheses

The general objective was to analyze the effect of the primary nursing model on the therapeutic self-care of older patients with multimorbidity during hospitalization.

Specific objectives:

To compare self-care scores between admission (T0) and discharge (T1).

To compare self-care scores between discharge (T1) and 30-day follow-up (T2).

To compare self-care scores between admission (T0) and follow-up (T2).

Hypotheses:

**H1.**  *There is a statistically significant difference in therapeutic self-care scores between groups at baseline (T0).*

**H2.**  *The increase in self-care scores from T0 to T1 is greater in the intervention group.*

**H3.**  *The increase in self-care scores from T1 to T2 is greater in the intervention group.*

**H4.**  *The increase in self-care scores from T0 to T2 is greater in the intervention group.*

### 2.3. Measures

Sociodemographic and clinical data were collected through a structured questionnaire and patient medical records. Variables included age, sex, education level, household and caregiver. Clinical data included reasons for hospitalization and the number and type of chronic conditions.

Therapeutic self-care (dependent variable) was assessed using the Therapeutic Self-Care Scale—European Portuguese version (TSCS-EP), validated by Cardoso et al. [20], with Cronbach’s alpha = 0.979. This instrument assesses the following areas of therapeutic self-care: management of the therapeutic regimen; ability to recognize symptoms or changes in health status, particularly those that may be indicative of complications; ability to perform normal activities; and ability to identify and implement strategies to adapt to the health condition. The scale consists of 12 items, with responses rated on a 6-point Likert scale ranging from 0 (“I don’t know/I can’t”) to 5 (“I know/I can”).

### 2.4. Data Collection

The principal investigator and unit head nurses collected data. Newly admitted, eligible patients were approached, informed, and consented. TSCS-EP assessments were completed at three time points: T0 (within 48 h of admission), T1 (on discharge), and T2 (30 days post-discharge, via telephone). Completed questionnaires were submitted in sealed envelopes with participant codes.

### 2.5. Data Analysis

Data were analyzed using SPSS^®^ version 28 for Windows (IBM, Chicago, IL, USA). Descriptive statistics were used for participant characterization. Categorical variables were presented as frequencies and percentages; continuous variables as means and standard deviations.

Levene’s test was used to test homogeneity of variances. Student’s *t*-test for independent samples compared group means. Chi-squared or Fisher’s exact tests were used for categorical comparisons [21]. Effect size was calculated using Cohen’s d [22].

An exploratory factor analysis (EFA) was performed to validate the psychometric properties of the TSCS-EP in the study sample, hereafter referred to as Therapeutic Self-Care Scale applied to People with Multimorbidity in Hospital (EATPPMH). The analysis followed standard criteria: KMO > 0.70, Bartlett’s test *p* < 0.001, Cronbach’s alpha > 0.70, communalities > 0.5, and factor loadings ≥ 0.4. Varimax rotation and eigenvalue > 1 were used. Correlations were interpreted as: low (0.2–0.39), moderate (0.4–0.69), high (0.7–0.89), and very high (0.9–1.0).

Multiple regression and logistic models were tested.

A significance level of 0.05 was adopted for all tests.

## 3. Results

**Figure 1:**
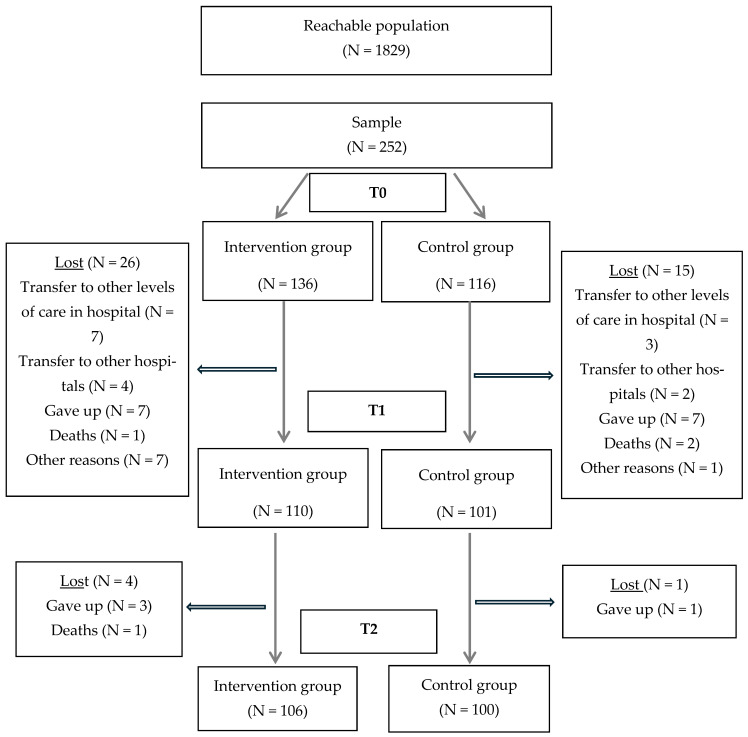
Flowchart of participant recruitment and retention.

A total of 252 participants were recruited at baseline: 136 in the intervention group and 116 in the control group. During hospitalization, 26 participants were lost in the intervention group and 15 in the control group. Between discharge and follow-up, an additional 4 participants were lost in the intervention group and 1 in the control group. In total, 106 patients in the intervention group and 100 in the control group completed the study, as illustrated in Figure 1.

Regarding participants’ sociodemographic characteristics, there were no statistically significant differences between the groups (*p* > 0.05). The mean age in both groups was approximately 75 years, and the majority were female. Most participants in the intervention group had primary education, while higher education was more frequent in the control group. In both groups, the majority lived with a spouse, who was also the primary caregiver. The average length of stay was two days longer in the intervention group than in the control group, and this difference was statistically significant (*p* = 0.020).

The reason for hospitalization showed a significant association with the distribution of participants between the two units (*p* < 0.001). Respiratory system diseases were the most frequent reason for admission in the intervention group, whereas musculoskeletal and connective tissue diseases, as well as skin and subcutaneous tissue diseases, were more common in the control group.

Most participants in both groups had two chronic diseases belonging to one of the selected study categories: cardiovascular disease, cancer, chronic obstructive pulmonary disease or diabetes. There was a significant association in the distribution of the number of chronic diseases between the groups (*p* = 0.002). Circulatory system diseases were the most prevalent chronic condition in both groups, and the majority of participants also presented other chronic conditions beyond those included in the study. Table 2 presents the sociodemographic and clinical characteristics of the participants.

### 3.1. Validation of the Psychometric Properties of the Therapeutic Self-Care Scale—Portuguese Version for People with Multimorbidity in Hospital (EATPPMH)

The EATPPMH was administered to the intervention group (N = 106) and the control group (N = 100). EFA was conducted to evaluate the psychometric properties of the scale at three points: admission, discharge, and follow-up, in both groups. The analysis used principal component analysis with varimax rotation.

No comparable factor structure was found across the groups and time points. The number of factors ranged from two to four, with a maximum explained variance of 75.5%. A three-factor solution was then forced for each time point to improve comparability, with the follow-up results showing the most stable solution and optimal variance explanation.

Kaiser-Meyer-Olkin values were 0.83 (intervention group) and 0.76 (control group), both considered good. Bartlett’s test of sphericity was significant in both groups (χ^2^_(66)_ = 1415.81, intervention; χ^2^_(66)_ = 873.49, control; *p* < 0.001), indicating appropriate correlations among items.

At follow-up, the three-factor model explained 81.7% of the variance in the intervention group and 72.5% in the control group:

Factor 1—Recognizing and managing signs and symptoms (4 items): explained 60.7% (intervention) and 49.5% (control) of variance.
Factor 2—Managing changes in health status (5 items): explained 13.7% (intervention) and 10.8% (control).
Factor 3—Managing medication (3 items): explained 7.2% (intervention) and 12.3% (control).

Internal consistency was very high in the intervention group (Cronbach’s α = 0.934) and high in the control group (Cronbach’s α = 0.895).

### 3.2. Therapeutic Self-Care at Admission, Discharge, and Follow-Up

Therapeutic self-care was assessed at three points: admission (T0), discharge (T1), and 30-day follow-up (T2). The mean scores for overall self-care and for each component were compared between the intervention and control groups at each time point. The two groups were also compared in terms of the difference in self-care between the various assessment points in the study.

At admission, the intervention group presented a significantly lower mean score for overall therapeutic self-care and for the “Recognizing and managing signs and symptoms” component, compared to the control group. No statistically significant differences were observed for the remaining components. The Cohen’s d effect size is low for all components. Results are detailed in Table 3.

Differences in self-care over time are shown in Table 4. From admission to discharge (T1−T0), the intervention group showed a significantly greater increase in overall self-care (*p* = 0.024) and in the “Recognizing and managing signs and symptoms” component (*p* = 0.037) compared to the control group.

Between discharge and follow-up (T2−T1), no significant differences were observed between groups in overall self-care or in any component.

Between admission and follow-up (T2−T0), the intervention group showed a significantly greater improvement in overall self-care (*p* = 0.008), and in the components “Recognizing and managing signs and symptoms” (*p* = 0.033) and “Managing medication” (*p* = 0.014). No significant differences were observed for the “Managing changes in health status” component. The Cohen’s d effect size is low for all components.

## 4. Discussion

The main aim of this study was to analyze the effect of the primary nursing model on therapeutic self-care in hospitalized older patients with multimorbidity. The findings revealed that the mean self-care score at baseline (T0) was significantly higher in the control group. At discharge (T1), the intervention group demonstrated a greater and statistically significant improvement in self-care compared to the control group. Although no significant differences were observed between the groups at follow-up (T2) relative to discharge, the change from admission to follow-up showed a significantly greater improvement in self-care in the intervention group.

Multiple regression and logistic models were tested using the sociodemographic and clinical variables. However, none of these models demonstrated statistical adequacy or acceptable explanatory power. They explained little of the data and were therefore not presented or discussed.

Sociodemographic characteristics were relatively homogeneous between groups, with no significant differences, except for the length of hospital stay, which was significantly longer in the intervention group by an average of two days. This may be related to the characteristics of hospitalization, as there was a significant association between reason for admission and hospital unit. It should be noted that the longer average length of stay observed in the intervention group could indicate more interactions with nurses and more time for health education. This may have contributed to the more positive self-care results. Cumulatively, respiratory and circulatory system diseases accounted for 45.3% and 40.5% of hospitalizations, respectively. These findings align with previous studies, such as Zhang et al. [23], who identified circulatory diseases as prevalent chronic conditions in hospitalized older patients with multimorbidity. The intervention group had a higher prevalence of respiratory and circulatory diseases. They may have been more receptive to the health education interventions led by nurses, which could explain the improvements in self-care observed. The intervention group also had a higher proportion of patients with three or four chronic conditions. This could have created scope for enhancing self-care scores.

The presence of multiple chronic diseases has been associated with greater difficulty in understanding health information and a higher risk of loss of functional independence [23,24]. At admission, participants in the intervention group had significantly lower mean self-care scores, particularly in the component “recognizing and managing signs and symptoms.” This may be partially explained by a lower socioeconomic status in this group, including lower education levels and professional activity in working age, factors known to negatively impact the development of self-care abilities [25]. These individuals may also face more difficulties in accessing health services, navigating the healthcare system, or obtaining necessary resources [26]. These findings confirm Hypothesis 1 (H1): a statistically significant difference existed in baseline global self-care scores between groups. To address the first specific objective, self-care was assessed at discharge and compared to admission. The intervention group demonstrated a significantly greater improvement in self-care, particularly in the “recognizing and managing signs and symptoms” component, confirming Hypothesis 2 (H2). These findings are consistent with Faessler et al. [27], who reported that patients receiving nurse-led care demonstrated significantly greater improvements in self-care than those receiving usual care. It should be noted that the analysis did not adjust for baseline differences between groups. This might have influenced the observed outcomes.

For the second objective, self-care was assessed at one-month post-discharge. Although the intervention group maintained higher self-care scores, the differences compared to the control group were not statistically significant, and Hypothesis 3 (H3) was not confirmed. These findings highlight the importance of continuity of care in the community. A systematic review analyzed 30 studies on the integration of primary healthcare after hospital discharge. The studies, which involved older people being followed up by primary healthcare after discharge from hospital, were compared with those in which no such follow-up took place. It was found that people who received follow-up care had a 32% lower risk of being readmitted to hospital within 30 days, and a 17% lower risk within six months [28]. Interventions that include self-management support, telephone follow-up, and medication reconciliation are particularly effective in reducing readmission rates in older adults [29].

The third objective compared the change in self-care from admission to follow-up between groups. The intervention group showed significantly greater gains, confirming Hypothesis 4 (H4). Notably, improvements were observed across all components in the intervention group, with significant gains in “recognizing and managing signs and symptoms” and “managing medication.”

The dropout rate was higher in the intervention group than in the control group. This may be partly explained by the fact that participants in the intervention group had a higher disease burden and a lower level of education [24].

Overall, the primary nursing model appears to have had a positive effect on therapeutic self-care among older patients with multimorbidity during hospitalization. However, the sustainability of these gains -seems to require coordinated community-based support. Nurse-led care models have shown potential to improve medication adherence, reduce emergency department use, and prevent hospital readmissions in this population [30].

### Recommendations and Limitations

For human resource and time reasons, participants were selected by convenience sampling rather than random sampling, which would allow for greater generalizability of the results, this is a limitation of the study.

No comparative analysis was performed between retained and lost participants, which may introduce bias and affect the generalizability of the findings.

Although multivariate models including sociodemographic, clinical, and hospitalization-related variables were tested, they did not demonstrate acceptable statistical adequacy or explanatory power. Consequently, adjusted analyses were not retained. As a result, baseline differences between groups (e.g., disease profiles, multimorbidity load, and length of stay) may have influenced the outcomes and confounded the observed effects.

In addition, it would be important to know the nurses’ opinions about the primary nursing model and its impact on nurse retention and satisfaction, which is a suggestion for future research.

## 5. Conclusions

The study suggests that the primary nursing model may be beneficial in improving self-care outcomes among older patients with multimorbidity during hospitalization. The components of self-care showing the greatest gains were recognizing and managing signs and symptoms and managing medication. However, these findings should be interpreted with caution because of baseline differences between groups, the loss of participants during the study, and the absence of adjusted analyses, which may have confounded the observed effects. Further studies using robust designs and appropriate adjustment for potential confounders are needed to confirm these results.

For any patient to be able to actively participate in the management of their health condition, they must have the skills to manage it effectively and efficiently. Nurses, by the nature of their interventions, whether through health education, empowerment or acquisition of self-care skills, are able to influence these patients’ health outcomes. At the same time, the nurse is a privileged interlocutor between the older patient’s with multimorbidity/family and the rest of the multiprofessional team.

Implementing this model may contribute to better health outcomes and reduced readmissions, emphasizing the nurse’s central role in empowering patients and coordinating multidisciplinary care.

## Figures and Tables

**Table 1:** Phases in the implementation of the primary nursing care model.

Phase	Intervention Unit	Time
Preparatory	Selection of primary nurses by the head nurse according to their skills.	1 week
	Specific training for the primary nurses.	4 weeks
	Training for the rest of the nursing team.	4 weeks
Implementation	Changing the care organization model	2 weeks
	Start recruiting patients	2 weeks later
Evaluation	Baseline on admission (T0)	Up to 48 h after admission
	On discharge from the unit (T1)	On the day of discharge
	Follow-up (T2)	30 days after discharge

**Table 2:** Comparison of sociodemographic and clinical characteristics at baseline between intervention and control groups.

		Intervention N = 106	Control N = 100	*p*-Value
Age (years), mean (SD)		76.4 (±7.4)	75.7 (±7.0)	0.498
Sex, N (%)	Male	63 (59.4)	53 (53.0)	0.352
	Female	43 (40.6)	47 (47.0)	
Education, N (%)	Basic	50 (47.2)	36 (36.0)	0.133
	Secondary	27 (25.5)	24 (24.0)	
	Higher	29 (27.4)	40 (40.0)	
Household N (%)	Spouse/partner	72 (67.9)	66 (66.0)	0.689
	Children	11 (10.4)	8 (8.0)	
	Lives alone	23 (21.7)	26 (26.0)	
Caregiver N (%)	Spouse/partner	51 (48.1)	48 (48.0)	0.334
	Children	26 (24.5)	32 (32.0)	
	Without a caregiver	29 (27.4)	20 (20.0)	
Length of stay, mean (SD)		8.7 (±7.0)	6.7 (±5.1)	**0.020**
Reason for hospitalization, N (%)	Circulatory system diseases	26 (24.5)	16 (16.0)	**<0.001**
	Respiratory system diseases	31 (29.3)	16 (16.0)	
	Digestive system diseases	12 (11.3)	11 (11.0)	
	Genitourinary system diseases	15 (14.2)	7 (7.0)	
	Diseases of the skin and subcutaneous tissue/Diseases of the musculoskeletal system and connective tissue	5 (4.7)	38 (38.0)	
	Other	17 (16.0)	12 (12.0)	
Number of chronic diseases selected for the study, N (%)	One disease	2 (1.9)	15 (15.0)	**0.002**
	Two diseases	56 (52.8)	45 (45.0)	
	Three diseases	32 (30.2)	33 (33.0)	
	Four diseases	16 (15.1)	7 (7.0)	
Type of chronic diseases, N (%)	Circulatory system diseases	57 (53.8)	54 (54.0)	0.172
	Diabetes Mellitus	9 (8.5)	16 (16.0)	
	Neoplasms	19 (17.9)	19 (19.0)	
	Chronic Obstructive Pulmonary Diseases	21 (19.8)	11 (11.0)	
Presence of other chronic conditions, N (%)	Yes	70 (66.0)	63 (63.0)	0.649
	No	36 (34.0)	37 (37.0)	

Note. The *p*-values in bold indicate *p* < 0.05. *t*-tests were applied to continuous variables and chi-square tests to categorical variables.

**Table 3:** Mean therapeutic self-care scores at baseline in the intervention and control groups.

Self-Care	Intervention N = 106 M	Control N = 100 M	*p*-Value	Cohen’s D
Global	4.06	4.29	**0.026**	−0.31
Recognizing and managing signs and symptoms	3.67	4.04	**0.017**	−0.33
Managing changes in health status	4.15	4.28	0.245	−0.13
Managing medication	4.44	4.67	0.053	−0.22

Note. The *p*-values in bold indicate *p* < 0.05. The *t*-test was applied.

**Table 4:** Differences in mean therapeutic self-care scores between assessment points in the intervention and control groups.

Self-Care		Intervention N = 106 M	Control N = 100 M	*p*-Value	Cohen’s D
Global	Between admission and discharge	0.21	0.01	**0.024**	0.32
	Between discharge and follow up	0.27	0.19	0.363	0.13
	Between admission and follow up	0.48	0.21	**0.008**	0.32
Recognizing and managing signs and symptoms	Between admission and discharge	0.36	0.09	**0.037**	0.29
	Between discharge and follow up	0.28	0.22	0.581	0.08
	Between admission and follow up	0.64	0.31	**0.033**	0.30
Managing changes in health status	Between admission and discharge	0.15	−0.04	0.065	0.26
	Between discharge and follow up	0.25	0.22	0.769	0.04
	Between admission and follow up	0.40	0.18	0.066	0.26
Managing medication	Between admission and discharge	0.10	−0.02	0.232	0.17
	Between discharge and follow up	0.30	0.13	0.058	0.27
	Between admission and follow up	0.40	0.11	**0.014**	0.34

Note. The *p*-values in bold indicate *p* < 0.5. The *t*-test was applied.

## Data Availability

Data available in a publicly accessible repository. The data presented in this study are openly available at https://data.mendeley.com/preview/pn2xz7×2cs?a=adb8279c-b9ed-40cb-8534-34bac2c85785 (accessed on 11 August 2025).

## Appendix A

**Table A1:** Trend Statement Checklist.

Paper Section/ Topic	Item No	Descriptor	Reported?	
			√	Pg #
**Title and Abstract**				
Title and Abstract	1	Information on how unit were allocated to interventions	x	1
		Structured abstract recommended	x	1
		Information on target population or study sample	x	1
**Introduction**				
Background	2	Scientific background and explanation of rationale	x	2
		Theories used in designing behavioral interventions	x	2
**Methods**				
Participants	3	Eligibility criteria for participants, including criteria at different levels in recruitment/sampling plan (e.g., cities, clinics, subjects)	x	3
		Method of recruitment (e.g., referral, self-selection), including the sampling method if a systematic sampling plan was implemented	x	3
		Recruitment setting	x	3
		Settings and locations where the data were collected	x	3
Interventions	4	Details of the interventions intended for each study condition and how and when they were actually administered, specifically including:	x	3
		○ Content: what was given?	x	3
		○ Delivery method: how was the content given?	x	3
		○ Unit of delivery: how were the subjects grouped during delivery?	x	3
		○ Deliverer: who delivered the intervention?	x	3
		○ Setting: where was the intervention delivered?	x	3
		○ Exposure quantity and duration: how many sessions or episodes or events were intended to be delivered? How long were they intended to last?	x	3
		○ Time span: how long was it intended to take to deliver the intervention to each unit?	x	4
		○ Activities to increase compliance or adherence (e.g., incentives)	NA	
Objectives	5	Specific objectives and hypotheses	x	4
Outcomes	6	Clearly defined primary and secondary outcome measures	x	4
		Methods used to collect data and any methods used to enhance the quality of measurements	x	4
		Information on validated instruments such as psychometric and biometric properties	x	4
Sample Size	7	How sample size was determined and, when applicable, explanation of any interim analyses and stopping rules	x	3
Assignment Method	8	Unit of assignment (the unit being assigned to study condition, e.g., individual, group, community)	x	3
		Method used to assign units to study conditions, including details of any restriction (e.g., blocking, stratification, minimization)	x	4–5
		Inclusion of aspects employed to help minimize potential bias induced due to non-randomization (e.g., matching)	x	4–5
Blinding (masking)	9	Whether or not participants, those administering the interventions, and those assessing the outcomes were blinded to study condition assignment; if so, statement regarding how the blinding was accomplished and how it was assessed.	NA	
Unit of Analysis	10	Description of the smallest unit that is being analyzed to assess intervention effects (e.g., individual, group, or community)	x	3
		If the unit of analysis differs from the unit of assignment, the analytical method used to account for this (e.g., adjusting the standard error estimates by the design effect or using multilevel analysis)	NA	
Statistical Methods	11	Statistical methods used to compare study groups for primary methods outcome(s), including complex methods of correlated data	x	4–5
		Statistical methods used for additional analyses, such as a subgroup analyses and adjusted analysis	x	4–5
		Methods for imputing missing data, if used	NA	
		Statistical software or programs used	x	4–5
**Results**				
Participant flow	12	Flow of participants through each stage of the study: enrollment, assignment, allocation, and intervention exposure, follow-up, analysis (a diagram is strongly recommended)	x	6
		○ Enrollment: the numbers of participants screened for eligibility, found to be eligible or not eligible, declined to be enrolled, and enrolled in the study	x	6
		○ Assignment: the numbers of participants assigned to a study condition	x	6
		○ Allocation and intervention exposure: the number of participants assigned to each study condition and the number of participants who received each intervention	x	6
		○ Follow-up: the number of participants who completed the follow-up or did not complete the follow-up (i.e., lost to follow-up), by study condition	x	6
		○ Analysis: the number of participants included in or excluded from the main analysis, by study condition	x	6
		Description of protocol deviations from study as planned, along with reasons	NA	
Recruitment	13	Dates defining the periods of recruitment and follow-up	x	3
Baseline Data	14	Baseline demographic and clinical characteristics of participants in each study condition	x	6–7
		Baseline characteristics for each study condition relevant to specific disease prevention research	NA	
		Baseline comparisons of those lost to follow-up and those retained, overall and by study condition	NA	
		Comparison between study population at baseline and target population of interest	x	6–7
Baseline equivalence	15	Data on study group equivalence at baseline and statistical methods used to control for baseline differences	x	7
Numbers analyzed	16	Number of participants (denominator) included in each analysis for each study condition, particularly when the denominators change for different outcomes; statement of the results in absolute numbers when feasible	x	8–9
		Indication of whether the analysis strategy was “intention to treat” or, if not, description of how non-compliers were treated in the analyses	NA	
Outcomes and estimation	17	For each primary and secondary outcome, a summary of results for each estimation study condition, and the estimated effect size and a confidence interval to indicate the precision	x	8–9
		Inclusion of null and negative findings	x	8–9
		Inclusion of results from testing pre-specified causal pathways through which the intervention was intended to operate, if any	NA	
Ancillary analyses	18	Summary of other analyses performed, including subgroup or restricted analyses, indicating which are pre-specified or exploratory	NA	
Adverse events	19	Summary of all important adverse events or unintended effects in each study condition (including summary measures, effect size estimates, and confidence intervals)	x	8–9
**Discussion**				
Interpretation	20	Interpretation of the results, taking into account study hypotheses, sources of potential bias, imprecision of measures, multiplicative analyses, and other limitations or weaknesses of the study	x	9–10
		Discussion of results taking into account the mechanism by which the intervention was intended to work (causal pathways) or alternative mechanisms or explanations	x	9–10
		Discussion of the success of and barriers to implementing the intervention, fidelity of implementation	x	9–10
		Discussion of research, programmatic, or policy implications	x	10–11
Generalizability	21	Generalizability (external validity) of the trial findings, taking into account the study population, the characteristics of the intervention, length of follow-up, incentives, compliance rates, specific sites/settings involved in the study, and other contextual issues	x	10–11
Overall Evidence	22	General interpretation of the results in the context of current evidence and current theory	x	10–11
